# Pantoea agglomerans Infection in Neonates: A Systematic Review of Case Reports

**DOI:** 10.7759/cureus.61704

**Published:** 2024-06-05

**Authors:** Christina Nanou, Maria Tzoraki, Dimitra Maria Apostolidi, Dimitra Metallinou

**Affiliations:** 1 Department of Midwifery, School of Health and Care Sciences, University of West Attica, Athens, GRC; 2 Medical School, National and Kapodistrian University of Athens, Athens, GRC

**Keywords:** neonatal intensive care unit, case series, case report, case study, infection, neonate, pantoea agglomerans

## Abstract

*Pantoea agglomerans*, a gram-negative bacterium, has emerged as an opportunistic pathogen, particularly within neonatal healthcare settings. Initially perceived as an innocuous environmental contaminant, *P. agglomerans* has been increasingly implicated in a spectrum of clinical infections, including neonatal sepsis and bacteremia. This systematic review conducts an in-depth analysis of the clinical cases published in 2003-2023, elucidating the multifaceted clinical presentations and therapeutic challenges associated with *P. agglomerans* infections in neonates. In total, 11 case reports and case series of 45 neonates from eight different countries were included. Most of the infected patients (57.8%) were reported in Asian countries (Sri Lanka, India, Kuwait) and involved preterm neonates (64.4%) with extremely low to low birth weight, and concurrent medical conditions including co-infections in a few of them (15.6%). Blood was the main culture source of the pathogen, accounting for 42 cases (91.1%) whereas clinical presentations in neonates exhibited considerable heterogeneity, encompassing common symptoms such as feeding difficulties, respiratory distress, fever, lethargy, and sepsis. Neonatal survival largely depended on the infection's origin and the timing of diagnosis. Considering antibiotic susceptibility as a criterion for treatment selection led to a 74% survival rate. Usually, a combination of antibiotics was used. There were 11 neonatal deaths reported, leading to an estimated mortality rate of 24.4%. We conclude that outbreaks within neonatal intensive care units underscore the importance of stringent infection control practices and heightened surveillance, especially considering the rapid disease progression noted in the included studies. Enhanced awareness and understanding of the clinical and microbiological characteristics of *P. agglomerans* infections are paramount for optimizing outcomes and reducing the burden of disease in neonatal populations.

## Introduction and background

*Pantoea*, a genus of gram-negative bacilli, exhibits distinctive characteristics including the absence of a capsule and spores, along with motility facilitated by peritrichous flagella. When cultured on nutrient agar, *Pantoea* typically forms smooth, translucent, and slightly raised colonies, often with yellow pigmentation. These bacteria are facultative anaerobes and test negative for the oxidase enzyme [1-4]. *Pantoea* demonstrates a versatile metabolic capacity, utilizing various carbon sources such as D-xylose, D-ribose, maltose, D-galactose, D-mannose, D-fructose, trehalose, and D-mannitol for energy production. The genus *Pantoea* encompasses 20 species [5,6], categorized into 13 distinct DNA hybridization groups. Among these species, *Pantoea agglomerans* and *P. dispersa* were the first identified. Several pathogenic species have been isolated from clinical specimens, including *P. conspicua, P. brenneri, P. septica, P. eucrina, P. gaviniae, P. calida*, and *P. ananatis*. In particular, *P. dispersa* accounts for approximately 5% of the cases while *P. agglomerans* is implicated in approximately 95% of cases, rendering it as the most frequently encountered species in neonatal infections [5].

The earliest documented instances of human infections attributed to *P. agglomerans* can be traced back to the early 1970s [7]. Formerly known as *Enterobacter agglomerans* or *Erwinia herbicola*, *P. agglomerans* exhibits a rod-shaped morphology and its biochemical features align with the *Enterobacteriaceae* family. It is ubiquitously distributed across various environmental niches, encompassing soil, water bodies, and plant surfaces [1,8,9]. Initially regarded as an incidental contaminant in environmental and agricultural settings, *P. agglomerans* has emerged as an opportunistic pathogen, particularly in neonatal healthcare environments, primarily through contamination of medical equipment and parenteral nutrition solutions [1,10]. Despite its historical perception as a benign commensal or saprophyte, *P. agglomerans* has been increasingly recognized as a causative agent in a spectrum of clinical infections, including neonatal sepsis and bacteremia [5,11,12]. 

Bloodstream infections are critical medical conditions that lead to higher mortality rates despite advancements in antibiotics and supportive care [13,14]. Gram-negative bacilli often cause these infections, and their impact is heavily dependent on the pathogen type and resistance to antibiotics [1], the patient's health status, the promptness and accuracy of diagnosis, and treatment and therapeutic interventions [15]. *P. agglomerans* infections, in particular, can present across a broad spectrum, including conditions such as synovitis, septic arthritis, osteomyelitis, bacteremia, peritonitis, cholelithiasis, endophthalmitis, endocarditis, dacryocystitis, urinary tract infections, meningitis, and brain abscesses [15,16]. Moreover, hypersensitivity reactions and allergic pneumonitis represent additional potential clinical sequelae [5,8,17]. Nevertheless, in neonatal populations, typical clinical indicators include respiratory distress, fever, lethargy, poor feeding, and manifestations of sepsis [18].

Infections caused by *P.agglomerans* represent a significant peril to neonatal populations, especially those afflicted by underlying medical complexities or premature birth, and have been associated with adverse outcomes such as neonatal death [5]. Cases of neonatal sepsis, bloodstream infections, and outbreaks within neonatal intensive care units (NICUs) have been previously documented, underscoring the necessity for heightened surveillance and implementation of effective management protocols [5,17-19].

In this systematic review of case reports, conducted according to a pre-established protocol, we aim to synthesize and analyze the findings described in individual case reports concerning *P. agglomerans* infection in neonates and thus furnish a comprehensive synopsis of the available evidence. To the best of our knowledge, no prior systematic review has been previously undertaken to address this particular issue. Consequently, it is envisaged that by elucidating prevailing trends and insights within clinical practice, coupled with disseminating accumulated knowledge on diagnostic modalities and therapeutic strategies, this review may serve as an instructive resource for healthcare professionals. Such an endeavor aims to enhance comprehension of the clinical complexities and prognostic trajectories encountered in neonates suffering from a *P. agglomerans* infection.

## Review

Materials and methods

**Figure 1:**
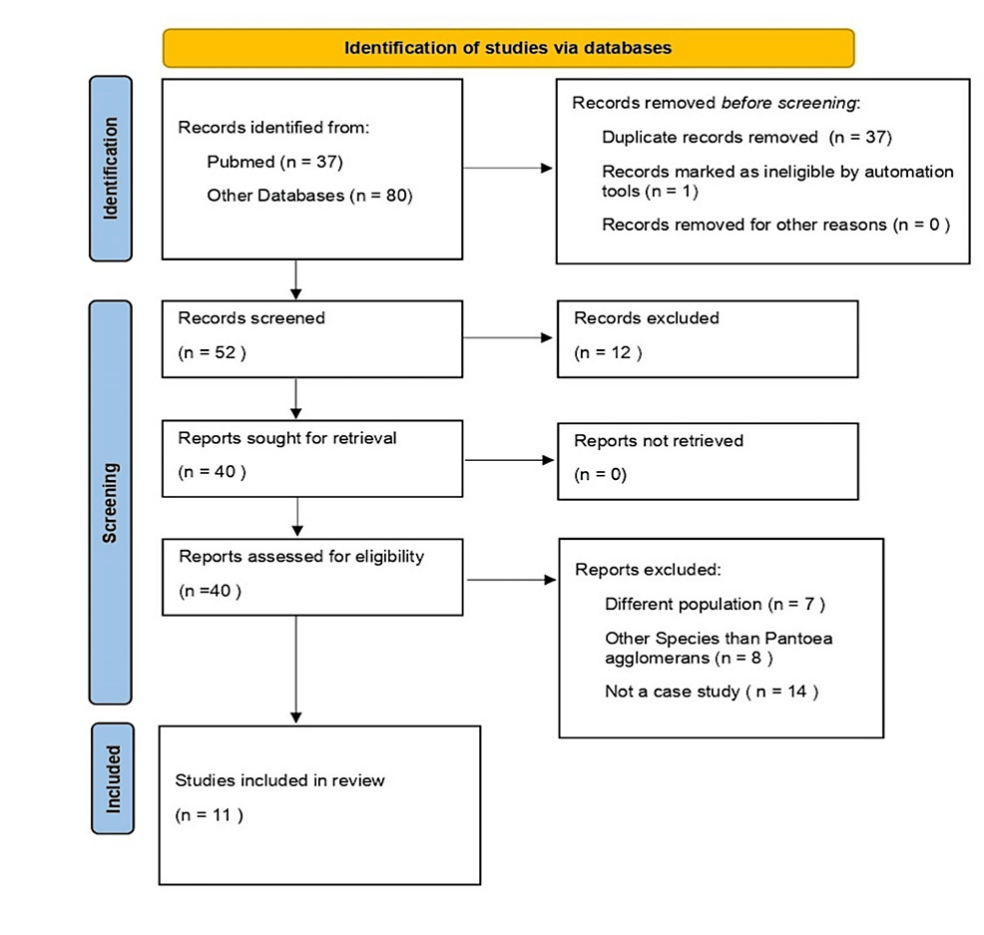
PRISMA flow diagram PRISMA: Preferred Reporting Items for Systematic Reviews and Meta-Analyses

A thorough literature review was conducted according to the Preferred Reporting Items for Systematic Reviews and Meta-Analyses (PRISMA) statement guidelines using the databases PubMed, Scopus, and Google Scholar to extract studies on *P. agglomerans* infections in neonatal populations. The flow chart, depicted in Figure 1, outlines the approach used for selecting studies to be included in the current systematic review.

The search strategy comprised the combination of Medical Subject Headings (MeSH) terms "pantoea agglomerans", "neonates", "infants", “neonatal intensive care unit”, "NICU", "infection", “case report”, “case series” and “case study”. The screening of the records was conducted based on title, abstract, and whether the full text met all the predetermined criteria as per the PICOS (Population, Intervention, Comparison, Outcomes, Study design). The search spanned from 2003 to 2023.

Due to the clinical heterogeneity of the condition, it was decided to encompass all cases focusing on *P. agglomerans* infection in neonates and providing detailed patient information, including demographics, gender, birth weight, gestational age, age in days at infection onset, culture source, comorbidities, clinical presentation, and treatment.

The articles were selected based on the following inclusion criteria: (a) papers reporting case studies/series/reports, (b) the study population included neonates, (c) infections solely attributed to *P. agglomerans*, excluding other species, and (d) cases that are published in peer-reviewed journals. The exclusion criteria included the following: (a) literature reviews, systematic reviews, meta-analyses, original research, and editorials, (b) studies not related to neonates, and (c) incomplete text. No restrictions were imposed on the language of publication. The investigation was conducted by two independent researchers (DMA and MT) and there were no discrepancies observed in the outcomes.

Results

In this systematic review, 11 papers that met the inclusion criteria were included. Among the selected papers, two lacked information regarding neonates' birth weights, one did not specify gestational ages nor reported clinical descriptions of the neonates. Case reports and case series concerning a total of 45 neonates from eight different countries are described. Most of the infected patients, 26 out of 45 cases (57.8%), were reported in Asian countries (Sri Lanka, India, Kuwait). The predominant subset of cases, 29 out of 45 (64.4%), pertained to preterm neonates characterized by extremely low to low birth weight (ranging from 630 g to 2500 g) and concurrent medical conditions, including co-infections in seven patients (15.6%).

Blood was the main culture source of the pathogen, accounting for 42 (91.1%) cases. *P. agglomerans* was isolated from a neck abscess, an anterior abdominal wall abscess, and the peritoneum in the remaining three cases. The clinical symptomatology of the infected neonates varied, encompassing respiratory distress for six patients (13.3%), disseminated intravascular coagulopathy for six cases (13.3%), and sepsis in 16 patients (35.6%), with some cases also showing signs of bradycardia and convulsions. Generally, the survival of the neonates was largely dependent on the origin of the infection and the timing of its diagnosis. Mustapha et al. [20], Aly et al. [18], and Lalas et al. [7] reported sporadic cases with unidentified sources of infection that appeared to follow a milder course and had a higher survival rate, likely due to the lower prevalence of virulent strains (Table 1). 

**Table 1:** Summary of articles included NM: Not Mentioned; CHD: congenital heart disease; DIC: disseminated intravascular coagulopathy; IUGR: intrauterine growth restriction; LBW: low birth weight; NEC: necrotizing enterocolitis; PDA: patent ductus arteriosus; PROM: premature rupture of the membranes; RDS: respiratory distress syndrome; MRSA:methicillin-resistant *Staphylococcus aureus*

Authors and Year	Country	N	Birth Weight (g)	Gestational Age (weeks)	Positive Culture Source	Age (days)	Hospitalization duration (d)	Antenatal Clinical Findings	Postnatal Clinical Findings/ Comorbitidies	Clinical presentation	Antibiotic Treatment	Outcome
Bergman et al., 2007 [8]	The Netherlands	3	3810	40	Blood	12	17	NM	CHD, hemothorax	Fever, hypotension, DIC	Amoxicillin/clavulanic acid/netilmicin (3 days) to vancomycin/cefotaxime	Death
			1795	29	Blood	5	8	PROM	RDS	Shock, DIC	Amoxicillin/gentamicin (5 days) to amoxicillin/cefotaxime to amoxicillin/clavulanic acid/tobramycin	Death
			630	28	Blood	20	34	IUGR, Fetal distress	RDS, coagulase negative *Staphylococcus* infection	Apnea, bradycardia, DIC	Amoxicillin/clavulanic acid/gentamicin to vancomycin/cefotaxime. Cefotaxime to meropenem	Death
Cruz et al., 2007 [21]	United States	5	NM	NM	Blood	16	NM	NM	Coartation of aorta, *Enterococcus faecalis* co-infection	NM	Aminoglycoside/broad-spectrum cephalosporin or ticarcillin-clavulanate (14-21 days)	Survived
			NM	NM	Neck abscess	17	NM	NM	Acinetobacter, MRSA co-infection	NM	Aminoglycoside/broad-spectrum cephalosporin or ticarcillin-clavulanate (14-21 days)	Survived
			NM	NM	Anterior abdominal wall abscess	7	NM	NM	*Klebsiella pneumoniae*, MRSA co-infection	NM	Aminoglycoside/broad-spectrum cephalosporin or ticarcillin-clavulanate (14-21 days)	Survived
			NM	25	Peritoneum	15	NM	NM	NEC, coagulase negative staph. infection	NM	Aminoglycoside/broad-spectrum cephalosporin or ticarcillin-clavulanate (14-21 days)	Survived
			NM	NM	Blood	24	NM	NM	Cardiomyopathy, *E. faecalis* co-infection, *Morganella* co-infection	NM	Aminoglycoside/broad-spectrum cephalosporin or ticarcillin-clavulanate (14-21 days)	Death
Aly et al., 2008 [18]	Kuwait	5	1500	30	Blood	10	20	NM	RDS	Lethargy, skin mottling, desaturation, bradycardia, jaundice, metabolic acidosis	Ampicillin/gentamicin (5 days) to cefotaxime/amikacin (10 days)	Survived
			1030	29	Blood	12	26	NM	RDS	Lethargy, skin mottling, bradycardia	Meropenem (14 days)	Survived
			815	28	Blood	17	31	NM	NEC	Abdominal distention, inactivity, attacks of apnea	Ampicillin/Amikacin (7 days) to meropenem (14 days)	Survived
			855	26	Blood	8	18	PROM	RDS, PDA	Lethargy, skin mottling, desaturation, bradycardia	Piperacillin/tazobactam (10 days)	Survived
			1020	27	Blood	11	15	NM	RDS, PDA	Lethargy, skin mottling, desaturation, bradycardia	Piperacillin/tazobactam (10 days)	Survived
Lalas and Erichsen, 2010 [7]	United States	1	1990	35	Blood	1	15	PROM, Foul smelling amniotic fluid	NM	Respiratory distress	Ampicillin/gentamicin to cefotaxime (14 days)	Survived
Segado-Arenas et al., 2012 [22]	Spain	1	750	32	Blood	8	19	Oligohydramnios, IUGR	Coagulase negative staph. infection	Respiratory distress, sepsis, fever, apnea, bradycardia, decreased peripheral perfusion, tachycardia, oligoanuria, DIC, pulmonary hemorrhage	Ampicillin/gentamicin (2 days) to meropenem/vancomycin	Death
Mahapatra et al., 2014 [12]	India	5	1500-2500	26	Blood	3	NM	NM	LBW	Lethargy, refusal to suck	According to sensitivity/NM	Survived
			1500-2500	27	Blood	7	NM	PROM, Foul smelling amniotic fluid	LBW	Abdominal distension	According to sensitivity/NM	Survived
			NM	32	Blood	5	NM	PROM, Foul smelling amniotic fluid	NM	Hypothermia, diarrhea	According to sensitivity/NM	Survived
			1500-2500	27	Blood	3	NM	NM	LBW	Hypothermia, diarrhea, irritability	According to sensitivity/NM	Survived
			NM	34	Blood	5	NM	Oligohydramnios	NM	Fever, iIrritability, poor suck	According to sensitivity/NM	Survived
Tiwari and Beriha, 2015 [9]	India	1	2900	40	Blood	2	> 14	PROM, Foul smelling amniotic fluid	NM	Respiratory distress, fever	Amikacin/vancomycin to meropenem (14 days)	Survived
Sengupta et al., 2016 [11]	India	1	2450	36	Blood	2	11	NM	LBW	Recurrent subtle generalized clonic seizures, thrombocytopenia	Cefotaxime/amikacin (2 days) to meropenem (10 days)	Survived
Senanayake et al., 2016 [23]	Sri Lanka	14	NM	12 preterm	Blood	NM	NM	NM	NM	Sepsis	According to sensitivity/NM	9 Survived
			NM	2 full-term	Blood	NM	NM	NM	NM	Sepsis	According to sensitivity/NM	5 Death
Gálvez-Cuitiva et al., 2018 [6]	Colombia	1	2340	35	Blood	1	17	NM	Meconium aspiration syndrome	Respiratory distress, sepsis, hypotension, DIC, pulmonary hemorrhage, thrombocytopenia, hepatic failure	Ampicillin (10 days)/gentamicin (5 days)	Survived
Mustapha et al., 2022 [20]	Nigeria	8	NM	39	Blood	27	8	None	None	Fever, convulsion, poor suck	Ampicillin/cloxacillin (Ampiclox)/gentamicin	Survived
			NM	38	Blood	6	NM	None	None	Fever, poor suck, jaundice, depressed primitive reflex	Ampicillin/cloxacillin (Ampiclox)/gentamicin	Survived
			NM	39	Blood	25	NM	None	None	Fever, convulsion, poor suck, depressed primitive reflex	Ampicillin/cloxacillin (Ampiclox)/gentamicin	Survived
			NM	39	Blood	4	20	None	None	Respiratory distress, fever, depressed primitive reflex	Ampicillin/cloxacillin (Ampiclox)/gentamicin	Survived
			NM	39	Blood	26	8	None	None	Convulsion, poor suck, depressed primitive reflex	Ampicillin/cloxacillin (Ampiclox)/gentamicin	Survived
			NM	38	Blood	16	8	None	None	Fever, poor suck, jaundice	Ampicillin/cloxacillin (Ampiclox)/gentamicin	Survived
			NM	39	Blood	12	11	None	None	Fever, jaundice	Ampicillin/cloxacillin (Ampiclox)/gentamicin (7 days) to ciprofloxacin (4 days)	Survived
			NM	38	Blood	2	5	None	None	Respiratory distress	Ampicillin/cloxacillin (Ampiclox)/gentamicin	Death

The authors indicated that when selecting a treatment plan, the susceptibility of the patient's sample to antibiotics was commonly considered, resulting in a survival rate of 74% (14 out of 19 cases), with a combination of antibiotics often being used (Table 2). The use of meropenem alone, although applied in just four out of the 45 recorded cases, resulted in exceptional outcomes with a survival rate of 100%. With regards to the cases included in this review, the estimated mortality rate for *P. agglomerans* infection in neonates, with 11 deaths occurring in 45 cases, stands at 24.4%.

**Table 2:** Classes of antibiotics used to treat neonatal Pantoea agglomerans infection NM: not mentioned

Treatment	Survival, n (%)	Death, n (%)
According to sensitivity/NM	14 (74%)	5 (26%)
Aminoglycoside + penicillin	8 (67%)	4 (33%)
Aminoglycoside + cephalosporin	5 (83%)	1 (17%)
Carbapenems	4 (100%)	0 (0%)
Carbapenem + clycopeptide	0 (0%)	1 (100%)
Cefotaxime	1 (100%)	0 (0%)
Penicillin + β-lactamase inhibitor	2 (100%)	0 (0%)

Clinical presentation

Clinical presentations of all included neonates are summarized in Table 3. 

**Table 3:** Clinical presentation of Pantoea agglomerans infection in neonates DIC: disseminated intravascular coagulopathy

Clinical Presentation	n (%)
Sepsis	16 (35.60%)
Fever	10 (22.20%)
DIC/Thrombocytopenia	7 (15.55%)
Respiratory Distress	6 (13.33%)
Bradycardia	6 (13.33%)
Poor Suck	6 (13.33%)
Lethargy	5 (11.11%)
Not Mentioned	5 (11.11%)
Skin Mottling	4 (8.88%)
Convulsions	3 (6.66%)
Pulmonary Hemorrhage	2 (4.44%)
Diarrhea	2 (4.44%)

Bergman et al. first identified the presence of *P. agglomerans* in 125 out of 6383 patients (2%) in a 24-bed level III NICU from 1994 to 2005 [8]. In this study, the authors described three cases of *P. agglomerans* infection in neonates who developed late-onset septicemia and subsequently died. The first patient was a full-term female neonate diagnosed with pulmonary atresia and an intact ventricular septum, treated with epoprostenol infusion and prophylaxis for endocarditis. On the 10th day, the planned cardiac surgery was postponed due to complications from a central venous catheter insertion, which resulted in a right-sided hemothorax and hypovolemic shock. To manage these issues, a chest tube was placed, and the neonate was administered fluids and packed red blood cells via a femoral line, stabilizing her condition. However, two days later, the neonate presented with fever, leukocytosis, and thrombocytopenia. She required intubation and ventilation, circulatory support, and exhibited symptoms of diffuse intravascular coagulation (DIC). Despite the initiation of amoxicillin/clavulanic acid and netilmicin, later switched to vancomycin and cefotaxime, *P. agglomerans* was detected in blood cultures. The patient’s respiratory and circulatory status deteriorated, leading to her demise on the 17th day of life. Cultures of parenteral nutrition were not performed. The persistent sepsis could have been due to a thoracic spinal abscess, which was discovered during the post-mortem examination.

The second patient, a very premature male neonate born at 29+6 weeks of gestation, was admitted to the NICU and received antibiotics for suspected septicemia [8]. However, by the fifth day, his condition rapidly deteriorated, with signs of DIC becoming evident. He required intubation, mechanical ventilation, and circulatory support. The antibiotic treatment was adjusted to amoxicillin and cefotaxime; yet, a day later, blood cultures confirmed the presence of *P. agglomerans*. Although cerebrospinal fluid (CSF) cultures were negative, the antibiotic regimen was expanded to include amoxicillin/clavulanic acid and tobramycin. The neonate was initiated on parenteral nutrition. Regrettably, the patient suffered intracerebral hemorrhage and passed away eight days after birth. The origin of the *P. agglomerans* infection was never identified. The third neonate, who was also premature, was admitted to the NICU and received continuous positive airway pressure (CPAP) support and parenteral nutrition, undergoing the same antibiotic treatment as the second patient for suspected septicemia. At 12 days of age, surveillance cultures identified gastrointestinal colonization with *P. agglomerans*. By day 18, the last female neonate exhibited apnea, bradycardia, and feeding issues. Her treatment was similar to that of previous patients, but her condition failed to improve within 48 hours. Subsequently, her central venous catheter was replaced, and cefotaxime was switched to meropenem. Despite extensive supportive care, her condition worsened, resulting in her death at 34 days old. No postmortem examinations were conducted in either case.

The same year, Cruz et al. reported on five neonatal cases, ranging in age from seven to 24 days, infected by *P. agglomerans* [21]. Each patient was treated with intravenous aminoglycosides for 14-21 days, combined with a broad-spectrum cephalosporin or ticarcillin-clavulanate. The initial case involved a 24-day-old female neonate who presented with cardiomyopathy and ultimately succumbed. Subsequently, a 16-day-old male diagnosed with aortic coarctation survived. Despite a co-infection with methicillin-resistant* Staphylococcus aureus* (MRSA), two other patients, aged 17 days and seven days, also survived. Additionally, a 15-day-old neonate who developed necrotizing enterocolitis (NEC) eventually recovered. *P. agglomerans* was isolated from blood, a neck abscess, an anterior abdominal wall abscess, and the peritoneum in each patient, respectively.

In 2008, Aly et al. reported five cases of premature neonates in Kuwait infected with *P. agglomerans *[18]. The first case was a premature neonate born at 30 weeks of gestation who presented with lethargy, respiratory distress syndrome, skin mottling, bradycardia, desaturation, jaundice, and metabolic acidosis on the 10th day of life. The initial treatment included intravenous ampicillin and gentamicin for five days, subsequently switching to cefotaxime and amikacin for an additional 10 days. The neonate ultimately survived. The second and third cases involved neonates born prematurely at 29 and 28 weeks, respectively. At ages 12 and 17 days, they showed initial signs of infection similar to the first case and were treated with meropenem for 14 days, both surviving. The fourth and fifth cases were premature neonates born at 26 and 27 weeks, respectively, developing the infection at eight and 11 days old. Like the earlier cases, they showed consistent symptoms; the 11-day-old also displayed jaundice and metabolic acidosis, similar to the first case. Both were treated with piperacillin/tazobactam for 10 days, leading to successful outcomes.

In 2010, Lalas and Erichsen reported the first documented case of a bloodstream infection caused by *P. agglomerans* in a nearly full-term, otherwise healthy neonate [7]. The case involved a female neonate born vaginally at 35 weeks of gestation, weighing around 2000 grams. Initially showing symptoms of tachypnea and chest retractions, the neonate was quickly transferred to the NICU. Blood cultures taken on the second day confirmed the presence of *P. agglomerans*, prompting a 14-day treatment with cefotaxime. The neonate was discharged from the NICU at 15 days old. A microscopic examination of the placenta showed signs of acute chorioamnionitis.

Another case in Spain documented by Segado-Arenas et al. involved a neonate weighing 750 grams, born at 32+2 weeks of gestation, with oligohydramnios and intrauterine growth retardation [22]. Although there were no initial signs of respiratory distress syndrome at birth, the neonate was prophylactically given antibiotics (ampicillin and gentamicin) and fluconazole. By the eighth day of life, the patient exhibited symptoms of late-onset sepsis, including fever, apnea-bradycardia episodes, and elevated acute phase reactants. Treatment with vancomycin began following the detection of coagulase-negative *Staphylococcus* in a blood culture. On the 19th day, the neonate developed respiratory distress and progressed to refractory shock, unresponsive to catecholamines. Meropenem was added to the treatment. Despite intensive care, this case developed refractory shock and pulmonary hemorrhage, leading to death, four hours later. *P. agglomerans* was identified in the blood culture, the parenteral nutrition culture tested negative, and no postmortem examination was performed.

Mahapatra et al. described five additional neonates who also presented with lethargy, poor feeding, and bradycardia, indicative of late-onset sepsis [12]. Four of these were male neonates born at home while one was a female born via cesarean section. Three of them were low birth weight and premature. None showed signs of pneumonia, bleeding disorders, or DIC. When growth signals were detected in the BACT/ALERT system (bioMérieux SA, Marcy-l'Étoile, France), subcultures were taken from the respective blood culture bottles onto MacConkey agar and blood agar. On the MacConkey agar, colonies that fermented lactose were visible, while large, faint yellow, translucent colonies appeared on blood agar, showing uniformly sized gram-negative bacilli. These isolates were further identified as *P. agglomerans* with a 98% probability using API 20E strips for *Enterobacteriaceae.* Antibiotic sensitivity testing showed susceptibility to most of the tested antibiotics, including piperacillin-tazobactam, amikacin, aztreonam, ciprofloxacin, meropenem, and cefotaxime, although resistance to aztreonam and cefotaxime was noted in two cases. Based on the sensitivity report, empirical therapy was adjusted, leading to positive outcomes in all cases.

Similarly, in India, Tiwari and Beriha reported the case of a four-day-old neonate, delivered vaginally at term, who was admitted to the NICU, 48 hours post delivery due to fever, tachypnea, and chest retractions [9]. The neonate exhibited respiratory distress with pneumonia observed on chest X-ray. Blood samples were collected aseptically for aerobic and anaerobic blood cultures, and empirical treatment with amikacin and vancomycin was initiated. Subsequent culture revealed *Pantoea *species with sensitivity to certain antibiotics, prompting a switch to meropenem, which was administered successfully for 14 days based on sensitivity results. Other laboratory parameters indicated elevated C-reactive protein (CRP) levels and leukocytosis. 

Furthermore, in 2016, Sengupta et al. reported on a two-day-old neonate exhibiting specific symptoms such as generalized clonic seizures and later thrombocytopenia [11]. *P. agglomerans* was isolated from the blood cultures. Successful treatment with Meropenem led to the neonate's stable condition and discharge on the 11th day.

An outbreak of bloodstream infection that occurred in the NICU at the Teaching Hospital Kandy in March 2010, in Sri Lanka, reported by Senanayake et al. in 2016, included a total of 55 neonates [23]. Initially, all blood tests for these neonates upon their entry into the NICU were negative for infection. However, blood cultures taken from 14 neonates two to three days after their admission to the NICU returned positive for *P. agglomerans*. Of these, 12 were preterm neonates and two were full-term. All isolated strains of *P. agglomerans* showed susceptibility to a range of antibiotics. This was the only report where environmental samples were also collected, though none of these showed evidence of *P. agglomerans* contamination. Despite appropriate antibiotic therapy, five of the 14 affected neonates died from sepsis, while nine responded well to the treatment. This incident constituted the largest reported outbreak of bloodstream infections caused by *Pantoea* species in a NICU setting.

In a recent report by Gálvez-Cuitiva et al., a premature female neonate, born at 35 weeks with meconium aspiration syndrome, was empirically treated with ampicillin and gentamicin for suspected early neonatal sepsis [6]. The patient suffered from multisystem failure, necessitating intensive care with hemodynamic and respiratory support. Laboratory findings showed leukopenia, increased CRP levels, and impaired liver function. Blood cultures identified a *P. agglomerans* infection. Following the completion of her antibiotic regimen, she was discharged in stable condition after 17 days and continued showing normal development without further issues during outpatient follow-up.

Mustapha et al., in 2022, reported eight isolated cases of *P. agglomerans* bacteremia among 94 patients in the neonatal unit of Ahmadu Bello University Teaching Hospital over a 10-month period [20]. The clinical presentation of these neonates was described as mild, featuring symptoms such as fever, jaundice, convulsions, and diminished primitive reflexes, which contrasted with the more severe symptoms typically documented by other researchers. All affected neonates were full-term, born between 38 and 39+3 weeks of gestation, had no comorbidities, and had not been subjected to any invasive procedures. Notably, seven out of the eight neonates maintained a normal platelet count, but the one with the highest procalcitonin level ultimately did not survive. The treatment regimen included ampicillin with cloxacillin and gentamicin, with ciprofloxacin added for the seventh patient, spanning hospital stays of eight to 20 days.

The key findings and significance of each study to the current review are clearly outlined in Table 4.

**Table 4:** Key findings of each study and their significance to the present review. DIC:  disseminated intravascular coagulation; PROM: premature rupture of membranes

Study	Key Findings	Limitations	Conclusion	Significance to Review
Bergman et al. [8]	Large sample over an extended period. Infections linked to contaminated parenteral nutrition and central venous catheters.	Only described the three fatal cases. Inconsistent testing of potential infection sources, including parenteral nutrition.	Higher virulence in bacteremia. Lack of response to appropriate antimicrobial therapy could indicate a hidden focus of infection.	Emphasizes the virulence and presentation with septicemia in neonates.
Cruz et al. [21]	Larger sample of pediatric cases over a longer period, mostly with penetrating trauma but not in neonates. Varied infection sources.	Most cases were not neonates. Retrospective design.	Uncommon cause of infection in children. Antimicrobial susceptibility similar to other Gram-negative rods.	Highlights the need for higher clinical suspicion for better treatment and prognosis.
Aly et al. [18]	Reported higher survival rates with mild infection courses.	Lack of long-term follow-up data. Single-center study, unidentifiable infection source.	*Pantoea agglomerans* can cause mild, non-specific, community-acquired infections in neonates, with a good prognosis when treated with appropriate antibiotics.	Stresses the importance of early diagnosis and management strategies in improving outcomes.
Lalas and Erichsen [7]	Spontaneous case in a full-term healthy neonate.	Limited sample diversity, incomplete control of possible infection sources.	Correlation with antenatal infection. Rare cause of vertically transmitted disease in neonates.	Emphasizes considering this pathogen in similar clinical scenarios and highlights the need for further research.
Segado Arenas et al. [22]	First case reported in Spain. Rare but serious neonatal sepsis. Testing of parenteral nutrition.	Limited sample, lack of autopsy.	*P. agglomerans* is rare but severe, with high mortality and often poor antibiotic response.	Underscores the need for stringent infection control measures.
Mahapatra et al. [12]	Five neonates with late-onset sepsis responded well to antibiotics. No signs of DIC or pneumonia.	Limited sample, incomplete source culture, does not refer to specific treatment.	Detection of *P. agglomerans* in neonatal sepsis can improve understanding of its pathogenesis and clinical management.	Highlights the occurrence and treatment of *P. agglomerans* in preterm neonates, stressing the importance of early diagnosis and appropriate antibiotic therapy.
Tiwari and Beriha [9]	First case in India, full-term neonate, vaginal swab performed, associated with PROM.	Single case report. Identification as Pantoea species, the authors did not specify further identification.	Early detection leads to favorable outcomes.	Supports that early detection results in favorable outcomes. Discusses vertical transmission due to colonization of the vagina.
Sengupta et al. [11]	Unusual severe clinical presentation.	Small sample, no follow-up.	Early diagnosis and appropriate treatment.	Supports that early detection results in favorable outcomes even with severe clinical presentation.
Senanayake et al. [23]	Large sample (14 out of 55 cases), report of environmental samples.	Unidentifiable source. Limited to a single NICU outbreak.	Despite negative environmental samples, antibiotic sensitivity was consistent. Death occurred in 1/3 of cases.	*P. agglomerans* may cause NICU outbreaks even with an unidentifiable source. Highlights the importance of strict infection control measures.
Galvez-Cuitiva et al. [6]	A case of early-onset neonatal sepsis. Highlights the rarity of non-healthcare-associated neonatal infections and high mortality in preterms.	Single case.	Early identification and appropriate antibiotic treatment are crucial for better outcomes.	Emphasizes considering *P. agglomerans* in neonatal sepsis cases and the need for further research on its prevalence and impact.
Mustapha et al. [20]	Identified sporadic cases with unidentified infection sources. High survival rate.	Small sample size, limited geographic scope.	Higher survival rates due to lower virulence strains.	Highlights the need for vigilance even in mild cases due to potential unidentified infection sources.

Discussion

In neonates, *Pantoea* infections can manifest through diverse transmission routes, with vertical transmission being infrequent and lacking definitive evidence. Common sites of colonization include the trachea, urinary tract, and intestinal tract [5,24]. Exogenous sources primarily precipitate infections, frequently associated with the presence of central venous lines. Notably, contaminated parenteral nutrition emerges as a significant reservoir of infection among neonates hospitalized in NICUs [17,19]. Outbreaks of *Pantoea*-associated bloodstream infections in NICUs have been linked to the administration of intravenous fluids, infant formula, blood products, and anesthetic agents [2,19]. As indicated by Segado-Arenas et al. [22], heightened microbial burden from intravenous product contamination often leads to poor clinical outcomes despite appropriate antibiotic therapy, as observed in cases akin to those detailed by Bergman et al. [8], characterized by elevated CRP, irreversible shock, and DIC upon diagnosis. Conversely, cases documented by Aly et al. [18] (demonstrating favorable outcomes) and Mustapha et al. [20] (marked by sporadic occurrences and unknown infection sources) were identified at earlier disease stages and exhibited enhanced survival rates. Sporadic occurrences of *P. agglomerans* are attributed to strains of bacteria with lower virulence and bacteremic load, often present in the environment and on hand flora, as noted by Pancholi et al. [25]. The variability in clinical outcomes among neonates with *P. agglomerans* infections may be attributed to differences in the timing of diagnosis, the virulence of the bacterial strain, and the source of infection. Enhanced surveillance of intravenous products and early detection of infections could mitigate the risk and improve survival rates in affected neonates.

Furthermore, the possibility arises that *P. agglomerans* infection may stem from exposure to colonizing bacteria present in the birth canal, subsequently disseminating to the amniotic fluid following premature rupture of the membranes (PROM). This hypothesis is supported by the well-documented role of other *Enterobacteriaceae* members, such as *Escherichia Coli*, in vertically transmitted diseases in newborns, notably contributing to early neonatal sepsis [7,9,12,18]. Additionally, reports from some mothers indicate the presence of "foul-smelling amniotic fluid" [7,9,12]. While definitive conclusions remain elusive, there is a tantalizing speculation that *P. agglomerans* could represent an unusual source of vertically transmitted disease in otherwise healthy neonates. Interestingly, the bulk of documented cases in the literature, predominantly involving adults and children, attribute thorn-related injuries or trauma in rural settings as the primary source of infection. However, scant instances exist regarding neonates, with limited indications of maternal rural background or any history of injuries or trauma. *Pantoea* infections inflict greater severity upon preterm and critically ill term neonates due to factors such as immune deficiency, heightened virulence, or elevated bacterial load, similar to immunocompromised patients [15,21,26]. Such infections frequently culminate in high mortality rates among neonatal populations [5,22].

The complex clinical manifestation of *P. agglomerans* infections in neonates points out the challenges inherent in diagnosing and managing these cases, given the wide spectrum of symptoms ranging from skin mottling and respiratory distress to septic shock [17]. Many neonates present pneumonia and DIC, which can advance to complications such as pulmonary hemorrhage and intraventricular hemorrhage, ultimately resulting in mortality [8,11,17,22]. This clinical profile contrasts sharply with the presentation observed in other pediatric and adult cohorts, where infections are commonly associated with penetrating trauma and abscesses, such as osteomyelitis and septic arthritis, typically resulting in full recovery [21]. These differences may be attributed to several factors such as the immature immune system of the neonates which makes them more susceptible to severe infections and rapid disease progression, as well as the different infection routes. In neonates, infections might stem from vertical transmission or contaminated medical equipment, whereas in older children and adults, infections are more likely due to external trauma and environmental exposure. In addition, neonates often have multiple medical complications or comorbidities along with prematurity, thus increasing their vulnerability to infections. These observations emphasize the critical importance of early detection and prompt intervention in neonatal *P. agglomerans* infections. The cases discussed here exemplify the rapid disease progression and grave outcomes associated with such infections, highlighting the imperative for timely and effective therapeutic measures.

Diagnostic protocols for *P. agglomerans* infection conventionally entail blood cultures, typically incubated utilizing systems such as the BACTEC System (Becton, Dickinson and Company, Franklin Lakes, New Jersey, United States) [12,18]. Given the established link between *Pantoea *species and contamination of parenteral nutrition, it is prudent to additionally culture parenteral nutrition in cases of early neonatal sepsis to expedite diagnosis. Within a 24-hour timeframe, gram-negative motile bacilli affiliated with the *Enterobacteriaceae* family are frequently discerned, often exhibiting yellow pigment production on blood agar [18,27]. Rapid detection with blood cultures is essential for timely intervention in neonatal infections. Subsequent biochemical assays, encompassing oxidase and urease testing, triple sugar iron agar assessments, as well as reactions involving arginine dihydrolase, ornithine decarboxylase, and lysine decarboxylase, may be conducted to further delineate identification. Verification of microbial identity can be accomplished using systems such as the Vitek-2 system (bioMérieux SA) or API 20E strips, offering a high likelihood of *P. agglomerans* identification [11,18]. Characteristic colonies indicative of *P. agglomerans* typically manifest following overnight incubation [27]. Confirmation of *P. agglomerans* infection typically involves the Vitek-2 system or conventional biochemical methodologies, which ascertain the presence of catalase-positive strains, oxidase-negative strains utilizing glucose, mannose, rhamnose, and arabinose, devoid of urease activity, and refraining from lysine or ornithine decarboxylation [18]. The use of advanced identification systems accompanied by evidence-based knowledge regarding the key characteristics of this pathogen, ensures a relatively quick and accurate diagnosis, thereby improving neonatal outcomes.

The management of *P. agglomerans* infections in neonates presents a formidable challenge owing to its propensity for resistance against a broad spectrum of antibiotics [6]. Available evidence points out the resistance of *P. agglomerans* to multiple antibiotic classes, encompassing early-generation penicillins, cephalosporins, fluoroquinolones, aminoglycosides, trimethoprim/sulfamethoxazole, and tetracyclines. In cases of pneumonia or bacteremia, particularly in extremely premature neonates with underlying comorbidities, carbapenems are advocated as first-line therapeutic agents [5]. Limited data suggests that monotherapy with meropenem has yielded promising outcomes, boasting a 100% survival rate. For uncomplicated infections limited to the genitourinary tract, aminoglycosides may be considered as the first-line therapy [5,6]. In cases where *Pantoea* strains exhibit resistance to carbapenems, trimethoprim/sulfamethoxazole stands as a potential alternative. However, it is important to note the necessity for vigilant monitoring of serum bilirubin levels and liver enzymes throughout the treatment regimen when utilizing trimethoprim/sulfamethoxazole in neonates [5]. Ultimately, personalized therapeutic approaches predicated on susceptibility testing results and clinical response remain paramount for the optimal management of neonatal infections.

Moreover, the potential origins of infection, such as contaminated parenteral nutrition solutions, highlight the critical importance of rigorous infection control protocols within NICUs [7]. The diverse reactions to antibiotic therapy noted in the cases discussed emphasize the necessity for personalized treatment strategies and careful monitoring of neonatal patients afflicted with *P. agglomerans* infections [6]. While certain neonates positively responded to antibiotic treatment, others faced deteriorating clinical conditions despite intensive management interventions. This variability highlights the multifaceted nature of *P. agglomerans* infections and underlines the paramount significance of tailored patient management.

Future developments in addressing P. agglomerans infections in neonates should focus on several key areas, including state-of-the-art diagnostic tools, personalized medicine, infection control innovations, and enhanced clinical surveillance. More specifically, the development and implementation of more sensitive, rapid, and specific diagnostic technologies are crucial. Innovations like point-of-care testing, molecular diagnostics, and next-generation sequencing could facilitate early and accurate identification of P. agglomerans infections, enabling prompt and targeted treatment [15,28,29]. Tailoring treatments to individual neonates based on genetic, environmental, and microbiological factors might also improve outcomes. Research into the genetic predispositions and immune responses of neonates could lead to personalized therapeutic strategies that are more effective and have fewer side effects [30]. Furthermore, investing in new infection control technologies and protocols in neonatal care units is essential. This includes improved sterilization methods, the use of antimicrobial surfaces, and the integration of real-time infection tracking systems [31,32]. Finally, implementing comprehensive surveillance systems that use artificial intelligence and machine learning to predict and detect outbreaks [33,34] of P. agglomerans infections in real-time seems an innovative approach that might help in swiftly managing and containing infections. By focusing on these areas and advocating for policy changes that support increased funding for neonatal infection research, improved healthcare infrastructure, and equitable access to advanced medical technologies, the incidence and impact of P. agglomerans infections in neonates may be reduced and ultimately lead to safer and more effective neonatal care practices.

## Conclusions

Addressing *P. agglomerans* infections in neonates demands innovative approaches and forward-thinking strategies in healthcare settings. This review of clinical case reports not only highlights the diverse manifestations and management complexities of these infections but also points to a pressing need for a paradigm shift in neonatal care. The profound impact on premature and medically fragile neonates necessitates a reevaluation of current infection control practices, emphasizing proactive prevention and rapid response measures. The rapid disease progression and severe outcomes observed underscore the critical importance of early detection and timely, targeted interventions.

Advancing diagnostic technologies and refining therapeutic approaches remain crucial, yet the persistently high mortality rates signal that more is needed. A transformative change in the healthcare culture toward greater vigilance, enhanced training for healthcare professionals, and integration of cutting-edge research is essential to combat these infections effectively. Ultimately, comprehensive population studies employing rigorous methodologies are pivotal to understanding the full scope and impact of *P. agglomerans* infections in neonates. These studies should inform the development of robust preventive strategies and innovative improvements in neonatal care protocols. By fostering a collaborative and interdisciplinary effort and leveraging technological advancements, we can significantly mitigate the risks posed by *P. agglomerans*, ultimately optimizing outcomes and safeguarding the health of our most vulnerable populations.
